# Telomere attrition, kidney function, and prevalent chronic kidney disease in the United States

**DOI:** 10.18632/oncotarget.20706

**Published:** 2017-09-08

**Authors:** Moshen Mazidi, Peyman Rezaie, Adriac Covic, Jolanta Malyszko, Jacek Rysz, Andre Pascal Kengne, Maciej Banach

**Affiliations:** ^1^ Key State Laboratory of Molecular Developmental Biology, Institute of Genetics and Developmental Biology, Chinese Academy of Sciences, Beijing, China; ^2^ Institute of Genetics and Developmental Biology, International College, University of Chinese Academy of Science, Beijing, China; ^3^ Nephrology Clinic, Parhon University Hospital, Grigore T. Popa University of Medicine and Pharmacy, Iasi, Romania; ^4^ Department of Nephrology and Hypertension with Dialysis Unit, Medical University of Bialystok, Bialystok, Poland; ^5^ Department of Nephrology, Hypertension and Family Medicine, Medical University of Lodz, Zeromskiego, Lodz, Poland; ^6^ Non-Communicable Disease Research Unit, South African Medical Research Council and University of Cape Town, Cape Town, South Africa; ^7^ Department of Hypertension, Chair of Nephrology and Hypertension, Medical University of Lodz, Lodz, Poland; ^8^ Polish Mother's Memorial Hospital Research Institute (PMMHRI), Lodz, Poland

**Keywords:** telomere length, kidney function, glomerular filtration rate, national health and nutrition examination survey, albumin-creatinine ratio, Gerotarget

## Abstract

**Background:**

Telomere length is an emerging novel biomarker of biologic age, cardiovascular risk and chronic medical conditions. Few studies have focused on the association between telomere length (TL) and kidney function.

**Objective:**

We investigated the association between TL and kidney function/prevalent chronic kidney disease (CKD) in US adults.

**Methods:**

The National Health and Nutrition Examination Survey (NHANES) participants with measured data on kidney function and TL from 1999 to 2002 were included. Estimated glomerular filtration rate (eGFR) was based on CKD Epidemiology Collaboration (CKD-EPI) equation. Urinary albumin excretion was assessed using urinary albumin-creatinine ratio (ACR). We used multivariable adjusted linear and logistic regression models, accounting for the survey design and sample weights.

**Results:**

Of the 10568 eligible participants, 48.0% (*n*=5020) were men. Their mean age was 44.1 years. eGFR significantly decreased and ACR significantly increased across increasing quarters of TL (all *p*<0.001). The association between TL and kidney function remained robust even after adjusting for potential confounding factors, but the association between TL and ACR was only borderline significant (β-coefficient= -0.012, *p*=0.056).

**Conclusion:**

The association of kidney function with a marker of cellular senescence suggests an underlying mechanism influencing the progression of nephropathy.

## INTRODUCTION

Telomere is a repeat of specific short sequences of nucleotides found at the end of chromosomes. The length of telomere is so considerable, hence telomere shortening has been correlated with various pathological disorders and processes such as biological aging, oxidative stress and inflammation [1–6]. The stability of telomere is vital for the conservation of genetic information and maintaining cellular stability [7–9]. Additionally, distortion of telomere can result in cellular abnormality and organ failure [10, 11].

There is great interest in studying telomere length in relation with kidney health [12, 13], but clinical and epidemiological studies remain scanty. The limited existing evidence suggest that patients with end-stage renal disease (ESRD) may have shorter telomere and accelerated telomere shortening compared with the general population [14, 15]. Data for subjects with chronic kidney disease (CKD), derived mainly from two studies of severe heart failure patients, also suggest a strong correlation between reduced kidney function and shorter telomere length (TL), even after adjustment for age [16, 17].

The mechanism through which creatinine is regulated by the kidney, and the relationship between kidney function and TL are not fully understood, while existing data have been controversial [18]. However, clarifying the association of kidney function with TL is imperative, to confirm if this pathway holds promise for CKD risk evaluation and/or reduction.

In this study we investigated the association of TL, a marker for biological age, with kidney function and prevalent CKD using data from the National Health and Nutrition Examination Survey (NHANES).

## RESULTS

Of the 10568 eligible participants, 48.0% (*n* = 5020) were men. The mean age was 44.1 years overall, 43.5 years in men and 44.8 years in women (*p* = 0.063). With regard to education 52.5% (*n* = 4022) of the participants had completed more than high school, 25.6% (*n* = 2178) had completed high school, while 21.7% (*n* = 3248) had completed less than high school. White (non-Hispanic) represented 70.4% (*n* = 4864) of the participants, African-Americans - 10.9% (*n* = 2103) and Mexican-Americans - 6.9% (*n* = 2690). Overall, 20.8% were current smokers (24.8% of the men and 16.6% of the women). The mean and standard error of mean (SEM) for the TL in the overall sample was 1.08±0.015 (1.07±0.014 in men and 1.08±0.016 in women).

The distribution of participants by stage of CKD based on eGFR and ACR levels was the following: CKD stage 1 - 65.3%, CKD stage 2 - 26.5%, CKD stage 3 - 7.1%, CKD stage 4 - 0.6%, and CKD stage 5 - 0.4%. Overall, 8.1 % of the participants had eGFR less than 60 ml/min/1.73m^2^. Table 1 shows the characteristics of the participants according to their status for CKD. The lipid profile including triglyceride, total cholesterol, high-density lipoprotein cholesterol, and low-density lipoprotein cholesterol, was better in participants without CKD than in those with CKD (all *p < 0.001*).

**Table 1:** Antrpometrical and clinical characters of subjects based on CKD status

Variables	CKD Status		
	CKD Positive	CKD Negative	*p*-value
**Body Mass Index (kg/m**^2^)	30.2±2.32	27.5±3.52	<0.001
**Fat-free mass (kg)**	55.4±3.25	54.7±2.86	0.096
**Fat mass (kg)**	29.1±4.12	25.4±1.43	<0.001
**Triglyceride (mg/dl)**	174.1±5.82	149.2±7.49	<0.001
**Low density lipoprotein cholesterol(mg/dl)**	135.1±1.46	110.3±3.56	<0.001
**Total cholesterol(mg/dl)**	210.1±0.95	179.5±1.82	<0.001
**High density lipoprotein cholesterol(mg/dl)**	44.6±0.76	52.9±0.82	<0.001
**Systolic blood pressure**	132.5±0.26	119.6±0.24	0.079
**Diastolic blood pressure**	72.8±1.02	64.1±0.98	<0.001
**Fasting blood glucose(mg/dl)**	112.3±2.86	98.6±2.41	<0.001
**C-reactive protein(mg/dL)**	0.63±0.01	0.40±0.02	<0.001
**Log Cystatin C (mg/L)**	0.48±0.02	0.16±0.01	<0.001
**Urea Albumin (ug/mL)**	296.5±4.12	23.9±2.82	<0.001
**Glomerular filtration rate (mL/min)**	40.3±0.69	112.3±0.41	<0.001
**Log Albumin-Creatinine Ratio (mg/dl)**	2.0±0.01	1.8±0.01	<0.001
**CKD: chronic kidney disease**			

The association age- and sex-adjusted cardiometabolic factors, kidney function tests across quarters of TL analyzed are summarized in Table 2. Mean body mass index, fat-free mass, fat mass, triglyceride, total cholesterol and C-reactive protein significantly decreased across increasing TL quarters (all *p < 0.001*), while high-density lipoprotein cholesterol significantly increased across increasing TL quarters (*p* < 0.001, Table 2). eGFR significantly decreased and ACR significantly increased across increasing quarters of TL (both *p* < 0.001).

**Table 2:** Age-, sex-adjusted mean of kidney function tests level across quartiles of telomere length

**Variables**	**Quarters of telomere length**				**P-**
	**Q1**	**Q2**	**Q3**	**Q4**	**trend†**
**TL, Mean ±SEM (T/S ratio)**	0.73±0.02	0.92±0.01	1.08±0.01	1.38±0.06	
**Body Mass Index (kg/m**^2)^	28.5± 0.18	28.1±0.19	27.8±0.42	26.4±0.21	<0.001
**Fat-free mass (kg)**	54.8±0.94	54.4±0.96	53.4±0.74	51.0±0.68	<0.001
**Fat mass (kg)**	26.9± 0.89	25.8±0.32	24.9±0.76	24.1±0.96	<0.001
**Triglyceride (mg/dl)**	150.7± 5.7	142.1±6.2	140.1±5.9	133.0±5.1	<0.001
**Low density lipoprotein cholesterol(mg/dl)**	123.0±1.95	124.1±1.49	122.0±1.29	124.1±1.46	0.615
**Total cholesterol(mg/dl)**	203.1±1.9	199.6±1.07	189.3±1.92	180.7±1.46	<0.001
**High density lipoprotein cholesterol(mg/dl)**	49.3±0.62	51.1±0.49	52.1±0.73	54.0±0.82	<0.001
**Systolic blood pressure**	122.0±0.68	121.3±0.66	122.5±0.39	122.8±0.54	0.169
**Diastolic blood pressure**	72.06 ±0.54	73.2± 0.40	73.8±0.62	72.4±0.31	0.069
**Fasting blood glucose(mg/dl)**	101.0±1.2	101.3±1.05	102.4±1.82	101.9±2.31	0.905
**C-reactive protein(mg/dL)**	0.53±0.02	0.46±0.01	0.40±0.01	0.32±0.03	<0.001
**Log Cystatin C (mg/L)**	0.90±0.01	0.083±0.01	0.80±0.02	0.74±0.02	<0.001
**Urea Albumin (ug/mL)**	44.9±5.36	39.5±8.68	32.8±7.36	27.4± 4.43	<0.001
**Glomerular filtration rate (mL/min)**	96.5±0.92	99.9±0.74	101.4±0.64	106.2±0.69	<0.001
**Log Albumin-Creatinine Ratio (mg/dl)**	3.16±0.03	2.96±0.03	2.43±0.03	1.95±0.02	<0.001

^a^ *P*-values for linear trend across quarters of telomere length. Variables were compared across quartiles of telomere length using analysis of variance (ANOVA) test .CKD: chronic kidney disease.

Further univariable and multivariable (age-, sex-, race-, smoking-, fasting blood glucose-, systolic and diastolic blood pressure-, body mass index-, and C-reactive protein) regression analysis were performed to examine the association of TL with kidney function (Table 3). Univariable models revealed that TL was negatively associated with urea albumin and ACR (both *p* < 0.001), and positively associated with serum creatinine and eGFR (both *p* < 0.001). In multivariable adjusted models, the association remained significant between TL and eGFR, and borderline significant between TL and Urea Albumin (β-coefficient = -0.012, *p* = 0.056). Logistic regression was used to determine the association between quartile of the TL and chance of CKD, however we have failed to find any significant association between quartile of the TL and odds of CKD neither in crude nor in adjusted (age-, sex-, race-, smoking-, fasting blood glucose-, systolic and diastolic blood pressure-, body mass index-, and C-reactive protein) models.

**Table 3:** Univariate and multivariate (age-, sex-, race-, smoking-, fasting blood glucose-, systolic and diastolic blood pressure-, body mass index-, C-reactive protein, diabetes and hypertension) of association between kidney function tests level and telomere length

Variables	Univariate		Multivariate	
	Β-coefficient	*p*-value	Β- coefficient	*p*-value
Urea Albumin (ug/mL)	-0.094	<0.001	-0.09	0.069
Log Cystatin C (mg/L)	-0.264	<0.001	0.141	<0.001
Glomerular filtration rate (mL/min)	0.302	<0.001	0.195	<0.001
Log Albumin-Creatinine Ratio (mg/dl)	-0.142	<0.001	-0.131	0.025

## DISCUSSION

In this large representative sample of adults Americans, eGFR decreased while urinary albumin excretion increased across decreasing TL quarters. These patterns were robust to adjustment for potential confounding factors. Although, these findings did not translate into significant association between TL and prevalent CKD, our study findings suggest that telomere shortening could be an independent predictor of deteriorating kidney function.

In accordance with our findings, recent systematic review proposed that shortening TL might be related with CKD prevalence/occurrence or declining kidney function, however this relation is probably balanced by the cellular telomere reparative process in those surviving longer with CKD, furthermore stated that Short TL was independently related with increased risk of prevalent micro albuminuria in diabetic men with CKD [19]. Furthermore, recent Japanese investigation among persons with increased cardiovascular risk, telomere length indicated a relationship of longer telomere length to better renal function [20].

However, Pykhtina etal stated that no associations have been found between telomere length and inflammatory markers and the levels of glomerular filtration rate, urea and creatinine except for albuminuria which is associated with telomere length [21].

In line with our study, previous reports have suggested that TL is shorter in patients with end-stage renal disease patients on dialysis compared with the general population. For example, in a study of 15 patients on haemodialysis and 15 age-matched controls, the authors found an accelerated telomere shortening in patients on dialysis [22]. Another study in 18 diabetic patients on dialysis and 20 controls found an inverse correlation between TL and length of time in dialysis [14]. A study of 42 haemodialysis patients found reduced telomerase activity compared with non-haemodialysis patients [15].

Interestingly, recent investigation conducetd by Karin Luttropp concluded that Telomere attrition after 12 months was significantly greater in patients with renal replacement therapy compared to dialysis patients in addition non-CKD patients had meaningfully longer telomeres than CKD patients [23].

regarding to the evidence from pre-ESRD and CKD patients, earlier studies in patients with heart failure and normal range kidney function have reported a strong correlation between shortening TL and declining kidney function, even after adjustment for age [16, 17]. In this regard, Pim et al, have evaluated association of TL with renal function in 610 patients with heart failure (aged 40 to 80 years), and found that age-and sex-adjusted TL decreased steadily across decreasing quarters of eGFR [16]. Another study by Wong et al, explored the association between TL and renal function in patients with chronic heart failure (*n* = 866, median age was 74) [17]. They reported that TL was associated with renal function, even after adjustment for age, gender, age at chronic heart failure onset, and severity of chronic heart failure [17]. In contrast, the Heart and Soul study which is longitudinal study of patients with stable coronary heart disease, found that kidney function was not independently associated with shortened TL or telomere shortening over 5 years [24]. However their findings should be considered with caution, considering the focus on relatively old predominantly subjects (mean age was 66.7), with stable coronary heart disease [24].

This study has several strengths. It is one of the largest studies of the association of TL with kidney impairment. Kidney impairment was assessed by both eGFR and proteinuria. The selection of participants was based on random sampling of the general population and therefore the results can be extrapolated to the general population. As the data collection was performed on all days of the week throughout the year in NHANES, the potential for selection bias is very low [25, 26]. The findings from our study should be considered in the context of some limitations. The cross-sectional nature does not allow inference about causality. We did not have available any repeated measure of TL with quantitative polymerase chain reaction in the same subjects after several follow-up years to elucidate temporality of these findings.

This study has important clinical and public health implications. Understanding the interplay between TL and kidney function is a necessary and important step toward any application of the resulting knowledge for public health policy and action. Moreover we knew that CKD may increase the risk of CVD as leading cause of the death which may could be explain by role of the TL [27–29]. Our study provides a comprehensive snapshot of the relationship of kidney function with TL at the national level in the US.

In conclusion, our findings provide further evidence on the association between TL and kidney function. The association of kidney function with a marker of cellular senescence suggests an underlying mechanism influencing the progression of nephropathy.

## MATERIALS AND METHODS

### Population

The NHANES are ongoing repeated cross-sectional surveys conducted by the US National Center for Health Statistics (NCHS). The NCHS Research Ethics Review Board approved the NHANES protocol and consent was obtained from all participants [30, 31]. Data collection on demographic, diets, and behaviours occurred through questionnaires administered during home visits, while anthropometrics and biomarkers data were collected by trained staff using mobile examination units [30, 32]. The interview consisted of questions on socio-demographic characteristics (age, gender, education, race/Hispanic origin, and health insurance) and questions on previously diagnosed medical conditions. More detailed information on the NHANES protocol is available elsewhere [30, 33, 34]. This study was based on analysis of data from the 1999-2002 NHANES cycles. Analyses were restricted to participants aged 18 years and older. Fasting blood glucose (FBG), total cholesterol (TC), low density lipoprotein cholesterol (LDL-C), high density lipoprotein cholesterol (HDL-C), triglycerides (TG) levels and telomere length were assayed using methods described in the NHANES Laboratory/Medical Technologists Procedures Manual [30, 35, 36]. Complete laboratory procedures for collection, storage, calibration and quality control of blood samples for determination of hsCRP and other inflammatory markers are available elsewhere. [37] Creatinine was measured by the Jaffe reaction and standardized by methods described previously [38]. A random urine specimen was collected from participants, and urinary creatinine was measured by the Jaffe rate reaction, urinary albumin was measured by solid-phase fluorescent immunoassay [39]. Albuminuria was measured by urinary albumin-creatinine ratio (ACR) [39]. Glomerular filtration rate [eGFR, (ml/min/1.73m^2^)] was estimated using the CKD Epidemiology Collaboration (CKD-EPI) equation. CKD was defined as eGFR less than 60 (ml/min/1.73m^2^) [39].

### Telomere measurements

Aliquots of purified DNA, isolated from whole blood using the Puregene (D-50 K) kit protocol (Gentra Systems, Inc., Minneapolis, MN, USA), were obtained from participants. TL assay was performed using the quantitative polymerase chain reaction method to measure TL relative to standard reference DNA (also known as the telomere-to-single copy gene (T/S) ratio) [35, 40]. Each sample was assayed 3 times on 3 different days. The samples were assayed on duplicate wells, resulting in 6 data points. Control DNA values were used to normalize between-run variability [40, 41]. Runs with more than 4 control DNA values falling outside 2.5 standard deviations from the mean for all assay runs were excluded from further analysis (6% of runs). For each sample, any potential outliers were identified and excluded from the calculations (2% of samples). The inter-assay coefficient of variation was 6.5%. The Centers for Disease Control (CDC) conducted a quality control review before linking the TL data to the NHANES data files.

### Statistical analysis

We conducted the analyses according to the CDC guidelines for analysis of complex NHANES data, accounting for the masked variance and using the proposed weighting methodology [42]. To investigate the association between TL and kidney function, univariable and multivariable (age-, sex-, race-, smoking-, fasting blood glucose-, systolic and diastolic blood pressure-, body mass index-, C-reactive protein, diabetes and hypertension) regressions were applied. Adjusted (age- and sex-) mean of cardiometabolic factors and kidney function were compared across quarters of TL using the analysis of co-variance (ANCOVA) with Bonferroni correction. Variables were compared by using analysis of variance (ANOVA) and *Chi-*square tests. All tests were two sided, and *p* < 0.05 used to characterise statistically significant results. Data were analysed using SPSS^®^ complex sample module version 22.0 (IBM Corp, Armonk, NY, USA).
